# Insights into the FMNAT Active Site of FAD Synthase: Aromaticity Is Essential for Flavin Binding and Catalysis

**DOI:** 10.3390/ijms21103738

**Published:** 2020-05-25

**Authors:** Ana Serrano, Sonia Arilla-Luna, Milagros Medina

**Affiliations:** 1 Department of Biochemistry and Molecular and Cellular Biology, Faculty of Sciences, and Institute of Biocomputation and Physics of Complex Systems (Joint Units: BIFI-IQFR and GBsC-CSIC), University of Zaragoza, E-50009 Zaragoza, Spain; anaserra1979@gmail.com (A.S.); arilun2@gmail.com (S.A.-L.); 2 Centro de Investigaciones Biológicas Margarita Salas, CSIC, Ramiro de Maeztu 9, E-28040 Madrid, Spain

**Keywords:** flavin biosynthesis, prokaryotic FAD synthase, eukaryotic ATP:FMN:adenylyltransferase, aromatic residues, isoalloxazine, binding, rational mutagenesis, isothermal titration calorimetry, steady-state kinetics

## Abstract

The last step in the biosynthesis of flavin adenine dinucleotide (FAD) is considered a target for the design of antimicrobial drugs because it is carried out by two non-homologous proteins in eukaryotic and prokaryotic organisms. Monofunctional FMN: adenylyltransferases (FMNAT) in Eukarya and FMNAT modules of bifunctional FAD synthases (FADS) in Prokarya belong to different structural families with dissimilar chemistry and binding modes for the substrates. In this study, we analyzed the relevance of the hydrophobic environment of the flavin isoalloxazine in the FMNAT active site of *Corynebacterium ammoniagenes* FADS (*Ca*FADS) through the mutational analysis of its F62, Y106, and F128 residues. They form the isoalloxazine binding cavity and are highly conserved in the prokaryotic FADS family. The spectroscopic, steady-state kinetics and thermodynamic data presented indicate that distortion of aromaticity at the FMNAT isoalloxazine binding cavity prevents FMN and FAD from correct accommodation in their binding cavity and, as a consequence, decreases the efficiency of the FMNAT activity. Therefore, the side-chains of F62, Y106 and F128 are relevant in the formation of the catalytic competent complex during FMNAT catalysis in *Ca*FADS. The introduced mutations also modulate the activity occurring at the riboflavin kinase (RFK) module of *Ca*FADS, further evidencing the formation of quaternary assemblies during catalysis.

## 1. Introduction

**Figure 1:**
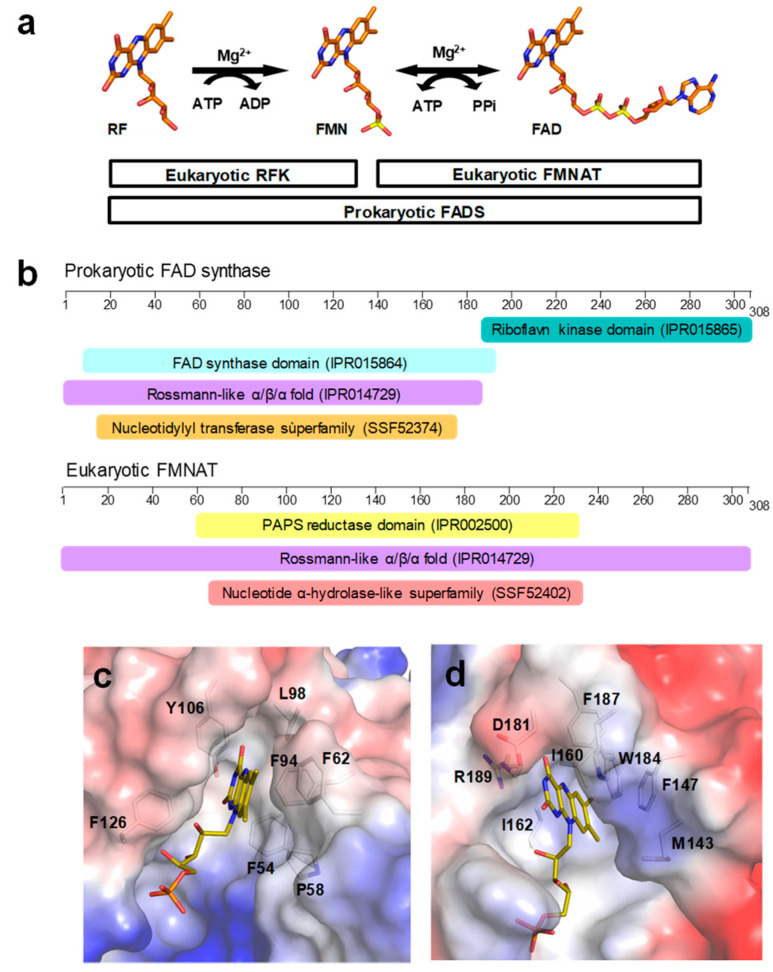
Sequence and structural features in FADSs. (**a**) Scheme of the flavin mononucleotide (FMN) and flavin adenine dinucleotide (FAD) biosynthetic pathway from riboflavin (RF) in eukaryotes and prokaryotes. (**b**) Classification of family domains for *Ca*FADS and *Cg*FMNAT as predicted by InterPro (https://www.ebi.ac.uk/interpro/). Electrostatic surface of the flavin mononucleotide adenylyltransferase (FMNAT) active site in (**c**) the FMNAT module of *Ca*FADS (PDB 2x0k) with flavin mononucleotide (FMN) docked according to Reference [12] and in (**d**) *Cg*FMNAT (PDB 3g5a) with FMN. Residues interacting with the ring are shown in sticks.

The biosynthesis of flavin mononucleotide (FMN) and flavin adenine dinucleotide (FAD) is considered a potential drug target for the search of new antimicrobials [1,2,3]. This is due to these flavin cofactors being essential for numerous flavoenzyme-catalyzed reactions, including protein folding, electron transport in the respiratory and photosynthetic chains, β-oxidation of fatty acids, nucleotide synthesis, or signal transduction among others [4,5]. All organisms synthesize FMN and FAD from riboflavin (RF) by the sequential action of two enzymatic activities, an ATP: riboflavinkinase (RFK) (EC 2.7.1.26), and an ATP: FMN adenylyltransferase (FMNAT) (EC 2.7.7.2) (Figure 1a). In prokaryotes, these two activities reside in a bifunctional enzyme named FAD synthase (FADS), consisting of an FMNAT module at the N-terminal and an RFK module at the C-terminal. By contrast, in most eukaryotes, two independent enzymes catalyze the production of FMN from RF, or of FAD from FMN [1,6]. The RFK module of prokaryotic FADSs shares sequence and structural homology with eukaryotic RFKs, but species-specific features have been recently reported [7,8,9,10]. By contrast, the FMNAT module differs from eukaryotic FMNATs (also known as FADSs) in sequence and structure. Although both adopt a Rossmann α/β/α topology, they belong to different protein families. While eukaryotic FMNATs with a 3-phosphoadenosine 5′-phosphosulfate (PAPS) reductase module belong to the adenine dinucleotide α-hydrolase-like superfamily [6,11], the FMNAT modules of prokaryotic FADSs belong to the nucleotidylyl transferase superfamily [7,9] (Figure 1b).

Structural information of eukaryotic FADSs is available for the yeasts *Candida glabrata* (*Cg*FMNAT), free and in complex with the FMN substrate, and an ATP analog as well as with FAD [6], and *Saccharomyces cerevisiae* (*Sc*FMNAT), which is, in this case, in a complex with FAD [11]. In *Homo Sapiens*, up to six FADSs isoforms (*Hs*FADS) have been identified as a result of alternative splicing of the same gene. The three longest isoforms contain, in addition to the PAPS reductase module, a molybdopterin binding-like module responsible for FAD hydrolase activity, not present in yeast FADSs. Two isoforms lack the PAPS reductase module while the last isoform discovered lacks the FAD hydrolase module. In addition, these isoforms also differ in subcellular location as well as in catalytic efficiencies and ions requirements [13,14,15,16,17,18]. There are no crystallographic structures available for any of these *Hs*FADSs, but some structural models have been built [17,19,20]. Lastly, a potential variety of mechanisms for regulation of FAD homeostasis in eukaryotic FADS might be occurring, as seen by the direct transfer of FAD from FADS to the client-apoproteins in human cells [21] or the product inhibition of the FMNAT activity reported in rat liver [22].

In prokaryotic FADSs, several members of the family, namely *Corynbacterium ammoniagenes* (*Ca*FADS), *Streptococcus pneumoniae* (*Sp*FADS), and *Listeria monocytogenes* (*Lm*FADS), are being thoroughly characterized [23,24,25,26]. Differential features among species are claimed to control FMN and FAD biosynthesis and homeostasis, such as stabilization of quaternary assemblies, redox requirements for RFK and FMNAT activities, and/or cooperativity in the binding of a substrate and products. Available crystallographic structures are those from *Thermotoga maritima*, *S. pneumoniae* (PDB 3op1), and *C. ammoniagenes* [27,28]. In addition, X-ray structures of the RFK module of *Ca*FADS in a complex with the products of this activity are also reported [9], and models for the interaction of substrates and/or products at the FMNAT module are built using docking and molecular dynamics simulations [12]. 

**Figure 2:**
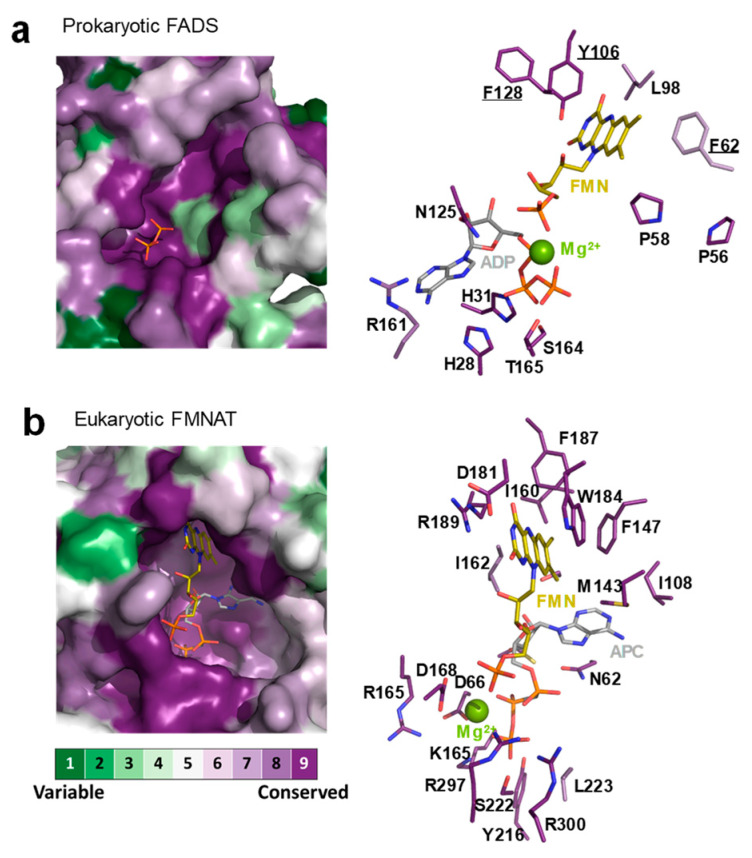
Sequence conservation at the FMNAT binding site in prokaryotic FADS and eukaryotic FMNAT families. Sequence conservation plotted on (**a**) *Ca*FADS (PDB 2x0k) and on (**b**) *Cg*FMNAT (PDB 3g5a) structures, showing active site for the FMNAT activity (left) and key residues for the binding of substrates/products highlighted as sticks (right). FMN and ATP:Mg^2+^ substrates in (a) docked according to Reference [12]. The conservation plot was produced by the ConSurf server [31] and drawn with PyMol [32].

Mutational analysis in *Ca*FADS have identified residues in its FMNAT module involved in catalysis and stabilization of ATP and FMN substrates. These studies have shown that: i) the competent binding of substrates is carefully driven by the side-chains of R161, S164, and T165 though interactions with the phosphates of FMN and ATP, ii) the binding site for competent interaction of the FMN isoalloxazine is shaped by conserved P56, P58, and L98 residues and iii) the adenine moiety of ATP is accommodated by H28 together with H31, while N125 approaches the reactant atoms of ATP and FMN acting as the FMNAT catalytic residue [29,30]. Moreover, while a hydrophobic environment coats the isoalloxazine cavity at the FMNAT module in *Ca*FADS, two charged residues, which are highly conserved in the family, are key for FMN binding to *Cg*FMNAT (Figure 1c,d and Figure 2). The hydrophobic cluster appears highly conserved in the prokaryotic FADS family (Figure 2a), which suggests a relevant role in the flavin catalytic competent orientation. In the present work, we studied the relevance of some of these hydrophobic residues, particularly F62, Y106, and F128, through their substitution by alanine (eliminating aromaticity), lysine (altering the residue nature), or tryptophan (increasing the residue volume while keeping aromaticity). The effects of these mutations in ligand biding and catalytic activity revealed the key role of the aromaticity at the FMNAT active site for the competent binding of the flavinic substrates/products, which is an essential feature for the adenylylation reaction to occur. These results, together with previous studies, provide a complete characterization of key residues at the binding site of substrates for FMNAT catalysis in the prokaryotic *Ca*FADS model. 

## 2. Results

### 2.1. Substitutions at F62, Y106, and F128 Produce Local Structural Changes

**Figure 3:**
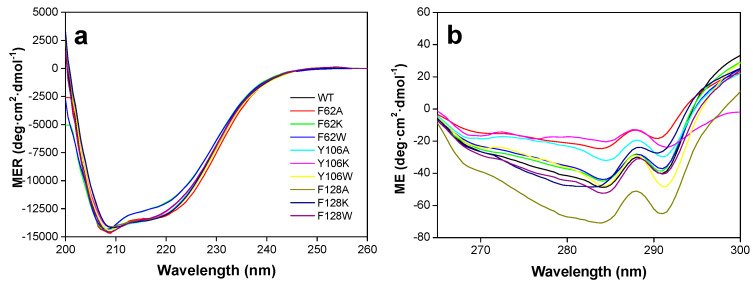
Conformation of *Ca*FADS variants. CD spectra (**a**) in the far-UV region (molar ellipticity per residue) and (**b**) in the near-UV region (molar ellipticity) for the different *Ca*FADS variants. Spectra were recorded respectively in 5 mM and 20 mM PIPES, 10 mM MgCl_2_, pH 7.0 at 25 °C.

All CaFADS variants were purified to homogeneity with similar expression levels to the wild-type (WT) enzyme, yielding ~10–15 mg of protein per g of wet cells (Figure S1a). Their correct folding was assessed by UV-visible spectrophotometry and circular dichroism (CD). UV-visible spectra showed a single maximum at 279 nm for all variants (Figure S1b). Nonetheless, while the determined extinction coefficient value for the WT enzyme was in accordance with the theoretical one (a difference of ~0.4%), values for the variants where, in general, their theoretical values are larger (up to 10% and 24% for Y106A and Y106K, respectively) (Table S1). The extinction coefficient values for the folded variants increased with regard to the WT protein. This effect was expected for those variants with an additional tryptophan, but such a feature was also observed for other substitutions. These observations indicate that the introduced mutations modulate the electronic environment of at least one nearby tryptophan. Far-UV circular dichroism (CD) spectra for the WT enzyme showed a minimum at 208 nm (α-helix, ~47%) and a shoulder at 222 nm (due to β-sheet, ~21%). In general, the variants showed similar far-UV CD spectra. Only variants at F62 showed a difference in the relative magnitude between the minima at 208 and 222 nm (Figure 3a), which suggested a decrease in the percentage of helix content (35%–40%) (Figure S2). Most of the mutations modify the magnitude of the near-UV CD spectra, which is in accordance with the modification of aromatic residues. This suggests changes in the local conformation of some tryptophan around the mutated residue (Figure 3b). Therefore, the introduced mutations do not alter the protein overall folding, but modulate the conformation of neighboring residues.

### 2.2. The Hydrophobic Core at the Isoalloxazine FMNAT site is Essential for FMNAT Activity and Influences the RFK Activity

**Figure 4:**
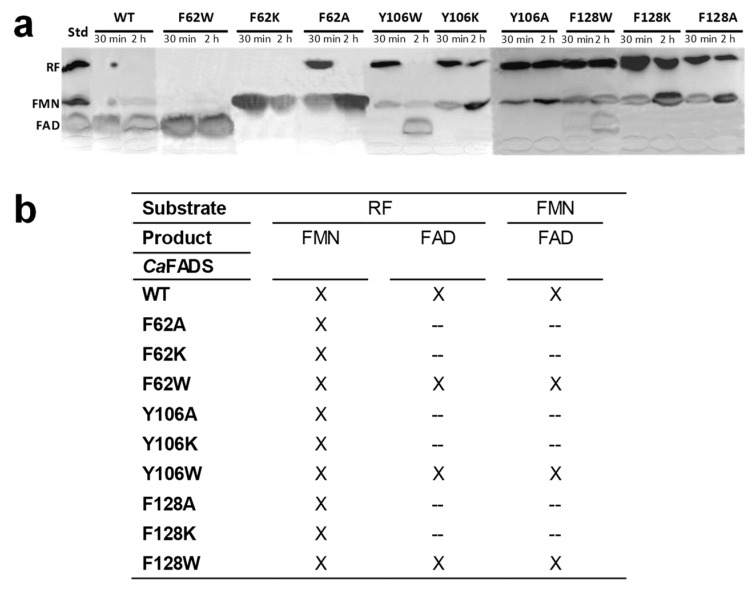
Profiles of the reactions of *Ca*FADS variants with its substrates. (**a**) Resolution by TLC of the products of the riboflavinkinase (RFK) activity of *Ca*FADS variants after incubation of reaction mixtures for 30 min and 2 h at 25 °C. (**b**) Summary of product formation after incubation of *Ca*FADS variants with RF or FMN at 25 °C during 2 h of reaction. Cross and hyphen indicate production and not detection, respectively, of the corresponding product. Reaction mixtures contained 200 nM of enzyme, 50 μM of RF or FMN, and 0.2 mM of ATP in 20 mM PIPES, 10 mM MgCl_2_, pH 7.0. Standard markers contain 50 μM of each of the flavins (RF, FMN, and FAD) incubated in similar conditions.

An estimation of the ability of the different variants to catalyze the transformation of RF and FMN was performed by incubating the substrates with the enzyme for 30 min or 2 h and resolving reaction products using thin layer chromatography (TLC) (Figure 4). 

According to the results, only the variants that kept aromaticity by introducing a tryptophan (F62W, Y106W, and F128W) were able to transform FMN into FAD. For the rest, no FMNAT activity was detected, even after 2 h of reaction. With regard to the RFK activity, all variants were able to produce FMN from RF. Nonetheless, RF was not totally consumed after 2 h of reaction for some variants, envisaging modulations in kinetic parameters by the introduced mutations. 

Steady-state measurements with dependences on concentrations of substrates were then performed to determine the kinetic parameters for RFK and FMNAT activities. The FMNAT parameters were only evaluated for F62W, Y106W, and F128W variants, which are the only ones showing FMN conversion into FAD (Figure 4). The introduction of a tryptophan at these positions barely affected the turnover rates for FMNAT. However, catalytic efficiency for FMN was reduced as shown by the increase in *K*_m_^FMN^ values (up to 11-times) (Table 1 and Figure S3a). Mutations had, if any, the opposite effect regarding the ATP substrate, decreasing *K*_m_^ATP^ when tryptophan substitutes for Y106 and F128. This suggests some mutations might favor ATP binding. All these results reveal the importance of the aromaticity provided by F62, Y106, and F128 side-chains for the FMNAT activity. 

We then determined the RFK kinetic parameters by quantifying FMN and FAD, which are products of the conversion of RF, as a function of time when varying substrate concentrations. The absence of FAD detection in the chromatographic profiles for variants containing alanine or lysine mutations confirmed their lack of FMNAT activity. Increasing concentrations of RF produced an important inhibition of the RFK activity for WT *Ca*FADS [34] with a ratio *K*_m_^RF^/*K*_i_ of 2.4. This inhibition has been attributed not only to the RF substrate inhibition but also to the reaction products, ADP and FMN, acting as competitive and uncompetitive inhibitors, respectively. Noticeably, such inhibition was considerably milder in the analyzed variants (with *K*_i_ >> *K*_m_^RF^), especially for F62A, F62K, and F128K, which showed *K*_i_ values considerably higher than that of the WT *Ca*FADS (Table 2 and Figure S3b, left). Moreover, all mutations decreased *k*_cat_ values but, with the exception of F62W and F128W, showed higher catalytic efficiencies for RF due to lower *K*_m_^RF^ values. The profiles for the RFK activity upon increasing concentrations of ATP followed a Michaelis-Menten model, as for the WT *Ca*FADS. In this case, the *k*_cat_ decreased for the variants, which resulted in lower catalytic efficiencies, except for F62A and F62K where increased in *k*_cat_/*K*_m_^ATP^ were observed as a consequence of a decrease in *K*_m_^ATP^ (Table 2 and Figure S3b, right). Therefore, the hydrophobic core of the isoalloxazine binding cavity of the FMNAT module of *Ca*FADS influences the RFK catalytic mechanism at the contiguous module of the enzyme.

### 2.3. The Properties of the FMNAT Isoalloxazine Binding Cavity Determines the Binding Mode of Flavinic Ligands at the FMNAT Module

**Figure 5:**
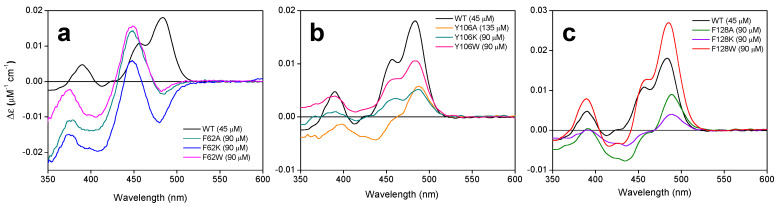
Internalization of the isoalloxazine ring of FMN in *Ca*FADS variants. Visible difference spectra elicited with (**a**) F62, (**b**) Y106, and (**c**) F128 *Ca*FADS variants (4–6 µM) upon titration with saturating FMN concentrations (indicated in parenthesis for each variant). In all panels, the difference spectrum of WT is included for comparison. Spectra recorded in 20 mM PIPES, 10 mM MgCl_2_, pH 7.0 at 25 °C.

**Figure 6:**
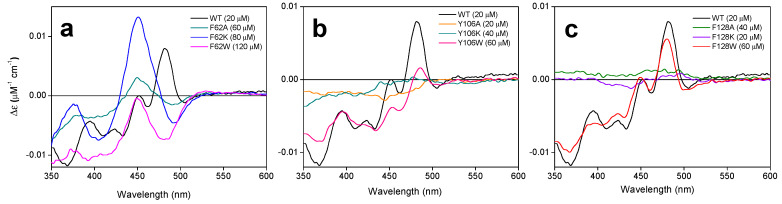
Internalization of the isoalloxazine ring of FAD in *Ca*FADS variants. Visible difference spectra elicited with (**a**) F62, (**b**) Y106, and (**c**) F128 *Ca*FADS variants (4–6 µM) upon titration with saturating FMN concentrations (indicated in parenthesis for each variant). In all panels, the difference spectrum of WT is included for comparison. Specstra recorded in 20 mM PIPES, 10 mM MgCl_2_, pH 7.0 at 25 °C.

The lack of FMNAT activity when these residues are substituted by alanine or lysine suggests that a right coupling of the isoallosazine to its cavity is required for the formation of the catalytic complex, but does not give us information about whether binding might be fully prevented. To better evaluate the effect of mutations on the interaction of *Ca*FADS variants with flavin cofactors, we first used difference spectroscopy in the absence of an adenine nucleotide. In these conditions, FMN and FAD are reported to exclusively bind at the FMNAT module of the enzyme [26], where the mutations are located. Incubation with flavins induced the appearance of different spectra for all variants (Figure 5 and Figure 6). 

In the case of FMN, variants at F62 produced the most important changes in the shape of the spectra with regard to the WT enzyme, with redshift of the spectrum and the maximum at 483 nm becoming a minimum (Figure 5a). However, differences in variants at Y106 and F128 were mainly observed in the magnitude of the signal, which were significantly lower for substitutions by alanine and lysine (Figure 5b,c).

Changes were much more relevant when evaluating the interaction of FAD (Figure 6). Additionally, in this case, a spectral redshift was observed for F62 variants and, with the exception of F62K, the magnitude of the signal was considerably reduced (Figure 6a). No difference spectrum was elicited by the titration with FAD of Y106A, Y106K, F128A, and F128K variants, and, only for F128W, the signal retained WT shape and intensity (Figure 6b,c). In any case, when binding is produced, all variants required higher FMN or FAD concentrations for achieving the maximal difference spectra signal. These observations indicate that the introduced mutations reduce the protein ability to internalize the isoalloxazine ring of the cofactor within its binding site, which fully prevents it when alanine or lysine substitute for Y106 or F128.

**Figure 7:**
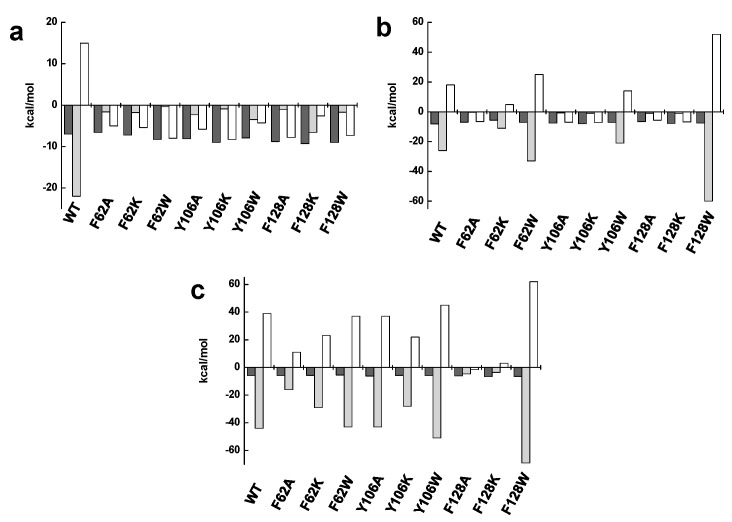
Thermodynamic contributions for ligand binding to *Ca*FADS variants. Thermodynamic dissections for the interaction of *Ca*FADS variants with (**a**) FMN, (**b**) FAD, and (**c**) ATP. The binding Gibbs energy (Δ*G*), enthalpy (Δ*H*), and entropy (-TΔ*S*) contributions to the binding are represented in dark grey, light grey, and white bars, respectively. Experiments were carried out at 25 °C in 20 mM PIPES, pH 7.0 for ATP, and in the presence of 10 mM MgCl_2_ for FMN and FAD.

The thermodynamic parameters for the binding of flavins to *Ca*FADS variants were then determined by isothermal titration calorimetry (ITC) (Table 3). In the assayed conditions, with 10 mM MgCl_2_, FMN and FAD bind at the WT *Ca*FADS FMNAT module with a 1:1 stoichiometry [26], with FAD binding six-fold stronger than FMN. By contrast, such behavior is inverted in the variants studied in this research, which increases affinity for FMN and decreases it for FAD (Table 3, Figure S4a,b,d,e). Affinity decreased for both flavins in F62A. In general, the thermodynamic profiles for the interaction of flavins also differed from that of the WT. Both the enthalpic and entropic contributions for FMN binding decrease in magnitude for all variants with the later one becoming favorable (Table S2 and Figure 7a). In the case of FAD, only substitutions by tryptophan kept a similar profile to the WT enzyme (showing an important increase in the magnitude of the enthalpic and entropic parameters for F128W) while the rest of the variants resembled the profile observed for the FMN interaction (Figure 7b). These observations confirmed that, while mutations at F62, Y106, and F128 do not prevent the binding of flavins at the FMNAT site of *Ca*FADS, they modify the binding mode of FMN and FAD as a consequence of the change in the hydrophobic properties of the isoalloxazine binding cavity.

### 2.4. The Hydrophobic Nature of F128 Modulates the Binding Mode of the ATP Substrate

Lastly, we also evaluated the effect of mutations on the binding of ATP. These experiments were carried out in conditions in which this nucleotide only binds to the FMNAT module in the absence of MgCl_2_. As for the WT, all the variants kept the same stoichiometry with one binding site, and, in general, similar affinity for ATP to that of the WT [26]. Nonetheless, the F128K and F128W substitutions produced an affinity increase (*K*_d_ values 2.5-fold and 3.4-fold lower than the WT) (Table 3, Figure S4c,d). The interaction of ATP was driven by a positive enthalpic contribution and an opposite entropic contribution for most variants, similarly to WT, but F128A and F128K variants changed the pattern (with an important decrease in the enthalpic contribution and the entropic contribution becoming favorable) (Figure 7c). These results suggest that, from the evaluated residues, only F128 somehow modulates the conformation of the ATP binding site.

## 3. Discussion

The FMNAT active site of *Ca*FADS is as an open cavity, coated by residues highly conserved in prokaryotic FADSs and with a highly hydrophobic cleft as a receptor of the isoalloxazine ring of the flavinic substrate (Figure 1 and Figure 2). In this case, we have particularly evaluated the key role of three hydrophobic and aromatic residues, F62, Y106, and F128, which conform to the isoalloxazine environment.

**Figure 8:**
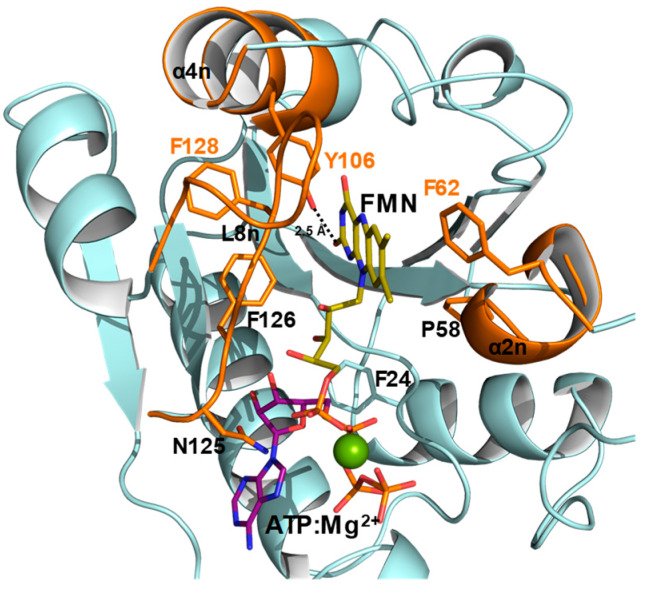
The FMNAT site of *Ca*FADS. Detail of the catalytic site for the FMNAT activity with FMN (yellow) and ATP:Mg^2+^ (magenta) substrates docked according to Reference [12]. P58, F62, Y106, and F128 (sticks), involved in FMN stabilization, and the structural elements in which they are located (cartoon) are highlighted in orange. F24, F126, and the catalytic N215 (located at loop L8n), which are involved in ATP stabilization, are also shown as sticks.

Substitutions at F62, Y106, and F128 do not alter the overall *Ca*FADS folding (Figure 3), but they highly compromise the ability of the enzyme to transform FMN into FAD. Only variants keeping aromaticity at these positions retain the ability to adenylylate FMN (Figure 4b and Table 1). Difference spectra and ITC data indicate that variants are able to bind substrates and products of the FMNAT activity (Figure 5 and Figure 6), but thermodynamic parameters clearly show a general decrease in the enthalpic contributions to the binding of flavinic substrates in favor of entropic contributions (Figure 7a,b, and Table S2). Therefore, changes in the aromaticity pattern at the FMNAT cavity hosting the isoalloxazine decrease the specificity of interactions with flavinic ligands, favoring their binding through unspecific interactions. Models for the interaction of FMN at the FMNAT site show the isoalloxazine siting in a hydrophobic pocket contributed by F54, P58, Y106, and F128, stabilized through a π-stack interaction between the flavinic ring and F62 and an H-bond between its C2 carbonyl and the hydroxyl of Y106 [12] (Figure 8). In agreement, the near-UV CD spectra indicate that mutations, independently of the nature of the introduced residue, have a strong effect in conformation of nearby aromatic residues, and due to the ability and mode of the isoalloxazine binding cavity to locate flavins (Figure 5a and Figure 6a). Some of the mutations will also considerably alter the surface electrostatic potential of the cavity, making it less hydrophobic, while the π-stacking and the H-bond would be clearly disrupted in variants at F62 and Y106, respectively. Altogether, these observations indicate that, despite the substitutions not preventing the FMNAT site of *Ca*FADS from FMN and FAD recognition, they have a clear deleterious effect in the competent assembly of their flavinic ring for catalysis. Moreover, for those variants that still show FMNAT activity, F62W, Y106W, and F128W, parameters indicate a slower rate for enzyme-substrate complex formation and/or for this complex turning over into a product during catalysis (Table 1 and Table 3). Lastly, only removal of aromaticity at F128 has an effect on the thermodynamic profiles for ATP binding (Figure 7c and Table S2). The side-chain of F128 does not point directly to ATP, but it is located at loop L8n next to residues N125 and F126, which, together with F24, form the binding cavity for the ribose moiety of ATP (Figure 8). The disposition of the side-chains of F126 and F128 in the WT protein promotes a π-stacking between them. Such stacking will be disrupted in F126A and F128K variants, where a different conformation can be envisioned for L8n to influence the ATP binding.

Comparison of the FMNAT active-site of *Ca*FADS with those of the eukaryotic enzymes carrying homologous functions revealed clear differences in the properties of the flavin binding cavities as well as in their binding mode (Figure 1 and Figure 2). While methyl groups of the isoalloxazine are expected to orient towards the solvent in *Ca*FADS [12], they are buried in eukaryotic enzymes. Thus, the environment around the isoalloxzine is more hydrophobic in the prokaryotic enzyme, while highly conserved charged residues contribute to the isoalloxazine site in the eukaryotic counterparts (Figure 1c and Figure 2). Some of these charged residues, such as D181 in *Cg*FADS and the equivalent D408 in *Hs*FADS, are crucial residues that contribute to the strong binding of the isoalloxazine of flavins, both as substrates and products [6,18,35]. In this way, the eukaryotic enzymes modulate their specific activity and the flavin homeostasis, making the release of the FAD product to the client-apoprotein the limiting step of the reaction [18,36].

**Figure 9:**
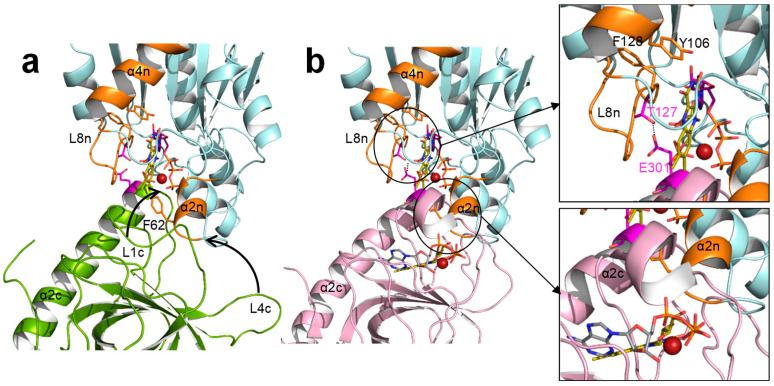
The head-to-tail conformation in the dimer-of-trimers assembly of *Ca*FADS. (**a**) Interface between RFK (green) and FMNAT (blue) modules of two contiguous protomers (PDB 2x0k) showing the open conformation of the RFK module. (**b**) Replacement in (a) of the RFK module by the closed conformation observed upon binding of the RFK activity products (pink) (PDB 5a89) and detail of the predicted T127-E301 H-bond [25], and the steric hindrance of structural elements in the closed RFK conformation of the trimeric structure. The required movement of loops for attaining this conformation are indicated with an arrow in (a).

In *Ca*FADS, flavin homeostasis has been related to the formation of a dimer-of-trimers assembly [35]. Mutations at F62, Y106, and F128 also modulate the kinetic parameters for the RFK activity (Table 2), which is in agreement with overall *Ca*FADS catalysis promoting the formation of such dimer-of-trimers [28,37]. The formation of this assembly is induced upon binding to the RFK module of ATP:Mg^2+^ as well as the products of its activity. In the formed dimer-of-trimers, the RFK module of one protomer sits on the FMNAT module of the contiguous protomer of the trimer in a head-to-tail organization that approaches the catalytic sites for FMN and FAD synthesis (Figure S5a). In this organization, the RFK cavity is partially closed by α2n and L4n of the FMNAT module of the contiguous protomer (Figure S5b) and the FMNAT cavity is covered by α1c of the adjacent protomer (Figure S5c). Moreover, crystallographic structures of the independent RFK module free and in complex with FMN and FMN:ADP revealed how binding of ligands at the RFK module induced conformational changes at loops L1c and L4c (Figure 9) [9]. Thus, an open binding site for RFK activity alternates with a closed active site upon binding of ligands. In the context of the trimeric assembly, the close contact between the RFK and FMNAT modules of contiguous protomers would imply that loop movements at the RFK module must be accompanied by structural changes at the FMNAT module, especially at α2n (Figure 9b, down), in order to avoid steric hindrances. The main effects observed by the mutations at the FMNAT module are decreases of substrate inhibition for the RFK activity of *Ca*FADS [34] which, in general, result in higher RFK catalytic efficiency. This effect is more remarkable for F62A, F62K, and F128K variants, which suggests that these mutations nearly prevent the formation of one or several of the different dead-end complexes related to inhibition of the RFK activity. Furthermore, F62 sits at the α2n that closes the RF binding site at the RFK module of the contiguous protomer. Its substitution by alanine or lysine must clearly affect the interaction of RF at the RFK binding site by decreasing the inhibition produced by this substrate. F128, although far from the RFK active site, is contiguous to T127, a residue that helps to stabilize the oligomeric structure through an H-bond with E301 of the RFK module in the contiguous protomer [25]. Changes at F128 could affect the conformation of L8n and, consequently, the stabilizing interaction T127-E301 (Figure 9b, up). The complexity of the catalytic mechanism of the bifunctional *Ca*FADS due to the formation of quaternary assemblies and due to the movements in structural elements during catalysis makes it difficult to assess the implication of these residues in RFK activity. However, according to the steady-state parameters obtained for this activity, its relevance is clear. 

In conclusion, F62, Y106, and F128 play a key role in the competent binding of FMN and FAD at the FMANT site of *Ca*FADS by providing a hydrophobic cavity that specifically recognizes their isoalloxazine rings. Together with a recent work involving residues P56, P58, and L98 at the entrance of the isoalloxazine cavity (Figure 2a) [30], this study also confirms that, in *Ca*FADS, the isoalloxazine binding mode is specific for the formation of the catalytic complex with the flavin and adenine substrates as well as their transformation into the reaction products. Despite its apparently open and broad active-site, the FMNAT module of prokaryotic *Ca*FADS confers a reaction specificity through a hydrophobic nature (provided by F62, Y106, and F128) and the shape (given by P56, P58, and L98) of its isoalloxazine binding site. In addition, this site’s results are different enough from eukaryotic FADSs to be considered a drug target for developing potential antimicrobials. Moreover, the results provide new evidence of the crosstalk between the RFK and FMNAT activities for regulating FMN and FAD biosynthesis by forming quaternary organizations, which is a mechanism not described for any other prokaryotic FADSs nor for eukaryotic FMNATs.

## 4. Materials and Methods 

### 4.1. Biological Material

pET28a-*Ca*FADS plasmids containing the F62A, F62K, F62W, Y106A, Y106K, Y106W, F128A, F128K, and F128W mutations were obtained from Mutagenex^®^. Proteins were over-expressed in BL21(DE3) *E. coli* cells and purified, as previously described [30,34] with 20% ammonium fractionation, which is followed by phenyl-sepharose and DEAE-cellulose chromatography and, then, dialyzed in 20 mM PIPES, pH 7.0. Protein purity was assessed by SDS-PAGE. 

### 4.2. Spectral Analysis

Spectroscopic measurements were performed in a Cary-400 spectrophotometer (Agilent Technologies, Santa Clara, CA, USA). The extinction coefficients in 20 mM PIPES, pH 7.0 at 279 nm (ε^279^) were calculated based on the method of Gill and von Hippel [38]. The UV-visible spectra of the folded enzymes and, after being denatured with guanidinium hydrochloride (6 M Gdn/HCl in 20 mM sodium phosphate, pH 6.5), were recorded. The extinction coefficient of the proteins in their denatured state, obtained from their amino-acid sequences, was used to calculate the concentration of the sample. Then, this concentration, taking into account the dilution during denaturation, was used to determine the ε^279^ in 20 mM PIPES, pH 7.0 from the Beer-Lambert law. 

Difference spectroscopy measurements were carried out in 20 mM PIPES, 10 mM MgCl_2_, pH 7.0 with ~4–6 µM *Ca*FADS, and the saturating concentrations of ligands were determined for each variant. 

CD spectra were recorded in a Chirascan spectropolarimeter (Appl. Phot. Ltd., Leatherhead, Surrey, UK) at 25 °C. Samples containing 5 µM *Ca*FADS in 5 mM PIPES, 10 mM MgCl_2_, pH 7.0, and 20 µM *Ca*FADS in 20 mM PIPES, 10 mM MgCl_2_, pH 7.0 were used in the far-UV CD (path length, 0.1 cm) or near-UV CD (path length, 0.4 cm), respectively. The spectra were analyzed to determine the percentage of secondary structural elements with the CDPro programs SELCON3 [39], CDSSTR [40], and CONTINLL [41]. 

### 4.3. Qualitative Detection of RFK and FMNAT Activities 

RFK and FMNAT activities were qualitatively assayed by separation of flavins from reaction mixtures by TLC on Silica Gel SIL-G-25 plates (20 × 20 cm, thickness 0.25 mm), as previously described [7,29]. The reaction mixtures containing 50 μM RF/FMN, 0.2 mM ATP, 10 mM MgCl_2_, and ~200 nM of *Ca*FADS in 20 mM PIPES, pH 7.0 were incubated for 30 min and 2 h at 37 °C. Reactions were stopped by boiling the preparations for 5 min. Flavin spots were visualized under UV light. 

### 4.4. Steady-State Kinetics Parameters for RFK Activities

The RFK and FMNAT activities were measured at 25 °C in 500 µL of 20 mM PIPES, pH 7.0 with 0.8 mM MgCl_2_ and 10 mM MgCl_2_, respectively. Reaction containing 0.5–55 µM RF or 0.2–150 µM FMN and 10–500 µM ATP were initiated by adding the enzyme (~20 nM). After 1 min of incubation at 25 °C, the reactions were stopped by boiling the mixtures for 5 min. The flavin composition in the supernatant was determined by using an Alliance high-performance liquid chromatography (HPLC) system (Waters, Milford, MA, USA) equipped with a 2707 autosampler and an HSST3 column (4.6 × 150 mm, 3.5 µm, Waters). This is preceded by a pre-column (4.6 × 20 mm, 3.5 µm, Waters). Flavins (FMN or FAD) produced from RF were quantified through their corresponding standard curves, as previously described [29]. 

The kinetic data (from triplicate experiments) obtained for one substrate at saturating concentrations of the second one (as nmol of flavin transformed per min) were fitted to the Michaelis-Menten kinetic equation (Equation (1)) or to the equation that describes the substrate inhibition in a bi-substrate mechanism [42] (Equation (2)).

(1) $$\frac{v_{0}}{\left[E\right]}=\frac{k_{\mathrm{cat}}\left[S\right]}{K_{m}+\left[S\right]}$$

(2) $$\frac{v_{0}}{\left[E\right]}=\frac{k_{\mathrm{cat}}\left[S\right]}{K_{m}+\left[S\right]\left(1+\frac{\left[S\right]}{K_{i}}\right)}$$

where *k*_cat_ is the catalytic constant, *K*_m_ is the Michaelis-Menten constant, and *K*_i_ is the inhibition constant. Errors for the *k*_cat_ and *K*_m_ values were of ±10% for Michaelis-Menten models while, in the case of substrate inhibition, errors in apparent *K*_m_ and *k*_cat_ (^app^*K*_m_ and ^app^*k*_cat_) increased with an inhibition constant (*K*_i_) getting closer to *K*_m_^S^. 

### 4.5. Isothermal Titration Calorimetry (ITC)

Measurements were carried out using a VP-ITC microcalorimeter (MicroCal LLC, Northampton, MA, USA) thermo-stated at 25 °C. Ligand (200 µM FMN or FAD and 300 µM ATP) and enzymes (~20 µM of *Ca*FADS) were dissolved in 20 mM PIPES, pH 7.0 (in presence of 10 mM MgCl_2_ for the flavins), and degassed prior to titration. Up to 28 injections of 4 µL of ligand were added to the sample cell (1.4109 mL) containing the enzyme and mixed via the rotating (1000 rpm) stirrer syringe. 

The association constant (*K*_a_), the enthalpy change (Δ*H*), and the stoichiometry (N) were obtained through non-linear regression of the experimental data to a home-derived model for one or two independent binding sites implemented in Origin 7.0 (OriginLab, Northampton, MA, USA) [7,29]. The dissociation constant (*K*_d_), the free energy change (Δ*G*), and the entropy change (Δ*S*) were obtained from basic thermodynamic relationships. Experiments were performed in duplicate or triplicate. Errors in the measured parameters (±15% in *K*_d_ and ±0.3 kcal/mol in Δ*H* and –TΔ*S* parameters) were taken as larger than the standard deviation between replicates and the numerical error after fitting analysis.

## Figures and Tables

**Table 1:** Steady-state kinetic parameters for the FMNAT activity of *Ca*FADS variants. Parameters obtained in 20 mM PIPES, 10 mM MgCl_2_, pH 7.0 at 25 °C, n = 3, mean ± SD.

*Ca*FADS	*k*_cat_ (min^−1^)	*K*_m_^FMN^ (µM)	*K*_m_^ATP^ (µM)	*k*_cat/_*K*_m_^FMN^ (min^−1^·µM^−1^)	*k*_cat/_*K*_m_^ATP^ (min^−1^·µM^−1^)
WT	39 ± 1	6 ± 1	43 ± 8	6 ± 1	0.9 ± 0.2
F62W	29 ± 1	42 ± 2	51 ± 7	0.7 ± 0.1	0.6 ± 0.1
Y106W	36 ± 1	12 ± 1	19 ± 2	3.6 ± 0.5	2.3 ± 0.2
F128W	26 ± 1	108 ± 13	9 ± 2	0.2 ± 0.1	2.9 ± 0.7

**Table 2:** Steady-state kinetic parameters for the riboflavinkinase (RFK) activity of *Ca*FADS variants. Parameters obtained in 20 mM PIPES, 0.8 mM MgCl_2_, pH 7.0 at 25 °C, n = 3, mean ± SD.

*Ca*FADS	*k*_cat_^app^ (min^−1^)	*K*_m_^RF^ (µM)	*K*_i_ (µM)	*k*_cat_^app^/*K*_m_^RF^ (min^−1^·µM^−1^)	*k*_cat_^app^ (min^−1^)	*K*_m_^ATP^ (µM)	*k*_cat_^app^/_m_^ATP^ (min^−1^·µM^−1^)
WT ^a^	408 ± 230	12 ± 3	4.9 ± 3.9	35 ± 22	155 ± 5	28 ± 4	5.5 ± 0.8
F62A	284 ± 31	2.5 ± 0.7	67 ± 22	1115 ± 33	127 ± 1	14 ± 1	9.3 ± 0.6
F62K	131 ± 3	0.2 ± 0.1	451 ± 131	534 ± 70	138 ± 5	18 ± 3	7.6 ± 1.2
F62W	151 ± 36	4.4 ± 1.8	20 ± 9	34 ± 16	92 ± 2	18 ± 2	5.2 ± 0.5
Y106A	143 ± 20	2.5 ± 0.8	34 ± 11	58 ± 19	53 ± 2	29 ± 4	1.8 ± 0.3
Y106K	148 ± 23	2.1 ± 0.7	23 ± 7	71 ± 26	54 ± 2	39 ± 6	1.4 ± 0.2
Y106W	42 ± 7	0.6 ± 0.3	34 ± 16	68 ± 37	21 ± 1	49 ± 7	0.4 ± 0.1
F128A	105 ± 20	2.6 ± 1.1	37 ± 18	40 ± 19	35 ± 1	30 ± 5	1.2 ± 0.2
F128K	68 ± 7	1.7 ± 0.5	98 ± 43	40 ± 12	51 ± 2	30 ± 4	1.7 ± 0.3
F128W	125 ± 24	4.3 ± 1.4	21 ± 8	29 ± 11	70 ± 2	38 ± 4	1.9 ± 0.2

^a^ Data from [25,33].

**Table 3:** Interaction parameters as determined for the binding of FMN, flavin adenine dinucleotide (FAD), and ATP to *Ca*FADS variants. Data obtained by ITC at 25 °C in PIPES 20 mM, pH 7.0, at the indicated MgCl_2_ concentrations, *n* = 3, mean ± SD.

	*K*_d_^FMN^ (μM)	*K*_d_^FAD^ (μM)	*K*_d_^ATP^ (μM)
MgCl_2_	10 mM	10 mM	—
WT	7.1 ± 0.4	1.2 ± 0.1	48 ± 7
F62A	15 ± 1	7.9 ± 0.7	44 ± 5
F62K	5.1 ± 0.3	75 ± 3	54 ± 9
F62W	0.8 ± 0.1	5.4 ± 0.1	74 ± 13
Y106A	1.2 ± 0.1	2.6 ± 0.1	31 ± 3
Y106K	0.3 ± 0.1	1.3 ± 0.1	58 ± 13
Y106W	1.7 ± 0.1	6.2 ± 0.2	44 ± 4
F128A	0.4 ± 0.1	14 ± 1	33 ± 3
F128K	0.2 ± 0.1	2.0 ± 0.1	14 ± 1
F128W	0.3 ± 0.1	2.5 ± 0.1	19 ± 1

## Supplementary Materials

The following are available online at https://www.mdpi.com/1422-0067/21/10/3738/s1, Figure S1: Conformation of *Ca*FADS mutants, Figure S2: Kinetic parameters for the FMNAT and RFK activities of the *Ca*FADS variants, Figure S3: Internalization of the isoalloxazine ring of FMN in *Ca*FADS variants, Figure S4: Internalization of the isoalloxazine ring of FAD in *Ca*FADS variants, Figure S5: Binding affinity of ligands to WT and F128K *Ca*FADS variants, Figure S6: Quaternary assembly of *Ca*FADS (PDB 2x0k), Table S1: Extinction coefficients for the *Ca*FADS variants, Table S2: Thermodynamic parameters for the interaction of *Ca*FADS variants with flavin and adenine nucleotides.

## Abbreviations

ATP	Adenosine 5′-triphosphate
CD	Circular dichroism
Gdn/HCL	Guanidinium hydrochloride
FAD	Flavin adenine dinucleotide, Riboflavin 5′-adenosine diphosphate
FADS	FAD synthase
FMN	Flavin mononucleotide, Riboflavin 5′-phosphate
FMNAT	ATP: FMN adenylyl transferase
HPLC	High performance chromatography
ITC	Isothermal titration calorimetry
PAPS	3-Phosphoadenosine 5′-phosphosulfate
PIPES	1,4-Piperazinediethanesulfonic acid
RF	Riboflavin
RFK	ATP: riboflavin kinase
TLC	Thin-layer chromatography
UV	Ultraviolet
WT	Wild-type
