# Supplementary material for: Insights into the FMNAT Active Site of FAD Synthase: Aromaticity Is Essential for Flavin Binding and Catalysis
Source: Int J Mol Sci. 2020 May 25;21(10):3738. doi: 10.3390/ijms21103738 (PMC7279473; doi:10.3390/ijms21103738)
Supplement: Supplementary file 1 [file ijms-21-03738-s001.pdf]

# Appendix A. Supplementary Material

# Insights into the FMNAT Active Site of FAD Synthase: Aromaticity is Essential for Flavin Binding and Catalysis

**Ana Serrano [1,2,†], Sonia Arilla-Luna [1,†] and Milagros Medina [2,*]**

[1] Department of Biochemistry and Molecular and Cellular Biology, Faculty of Sciences, and Institute of Biocomputation and Physics of Complex Systems (Joint Units: BIFI-IQFR and GBsC-CSIC), University of Zaragoza, E-50009 Zaragoza, Spain; anaserra1979@gmail.com (A.S.); arilun2@gmail.com (S.A.-L.)

[2] Centro de Investigaciones Biológicas Margarita Salas, CSIC, Ramiro de Maeztu 9, E-28040 Madrid, Spain

* Correspondence: mmedina@unizar.es

† These two authors have equally contributed to the study.

# SUPPLEMENTARY FIGURES

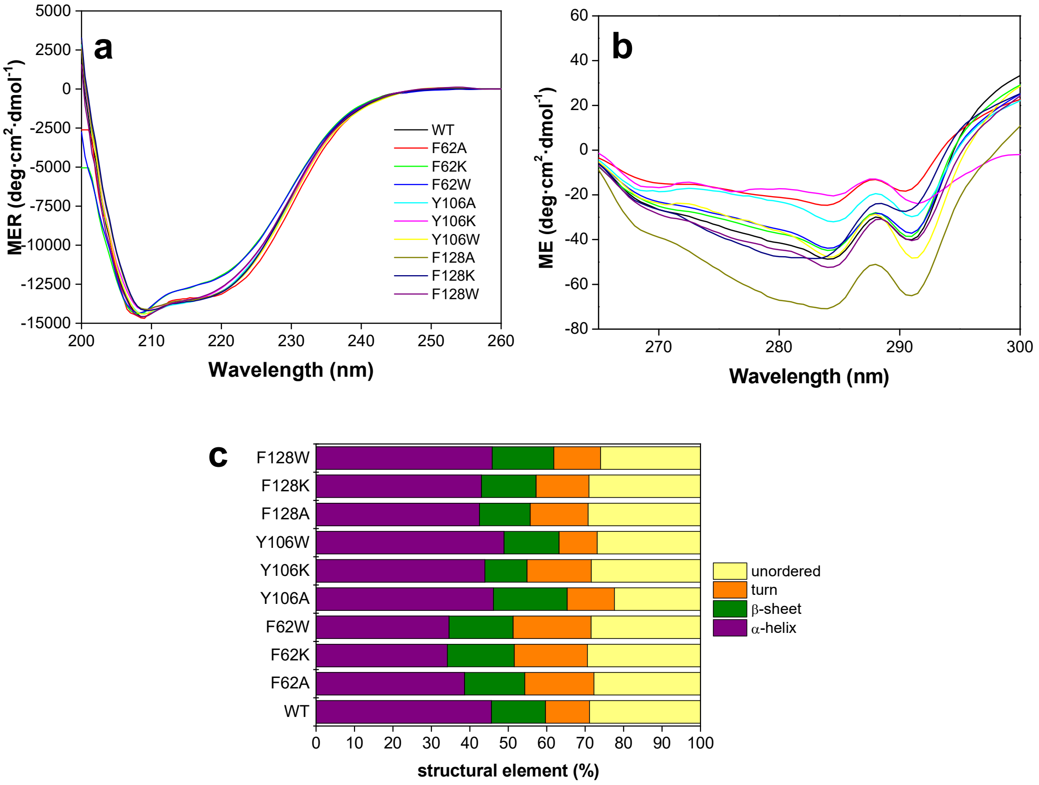


**Figure S1.** Conformation of *Ca*FADS mutants**.** CD spectra (molar ellipticity *per* residue) (**a**) in the far-UV region and (**b**) in the near-UV region for the different *Ca*FADS variants. Spectra were recorded respectively in 5 mM and 20 mM PIPES, 10 mM $MgCl_2$, pH 7.0 at 25 °C. (**c**) Percentage of secondary structure elements of the different *Ca*FADS variants estimated from CD spectra. Data correspond to the mean of estimations obtained with the CDPro softwares, SELCON3, CDSSTR and CONTINLL.

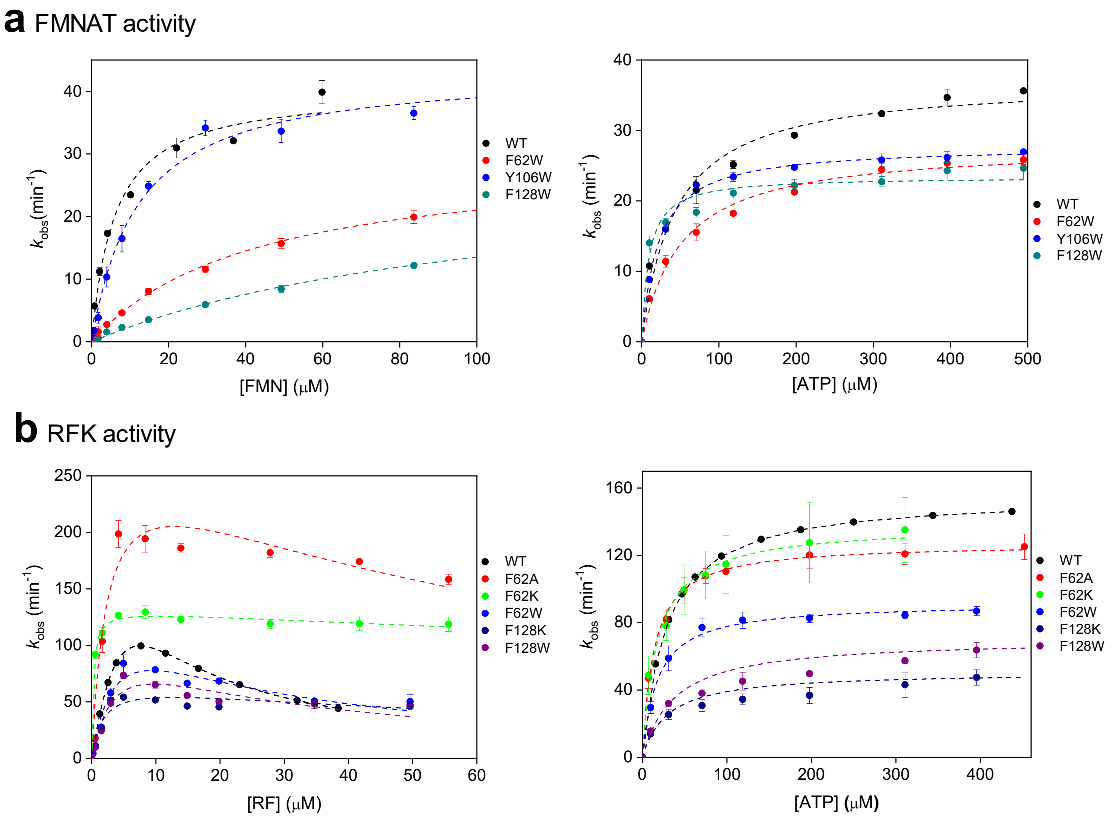


**Figure S2.** Kinetic parameters for the FMNAT and RFK activities of the *Ca*FADS variants. Steady-state rates for FMNAT (**a**) and RFK (**b**) activities of selected variants as a function of the flavinic substrate, FMN or RF, (*left*) and of the ATP substrate (*right*). Kinetics measured in 20 mM PIPES, pH 7.0, with 10 mM $MgCl_2$ for FMNAT activity and 0.8 mM $MgCl_2$,for RFK activity at 25 °C.

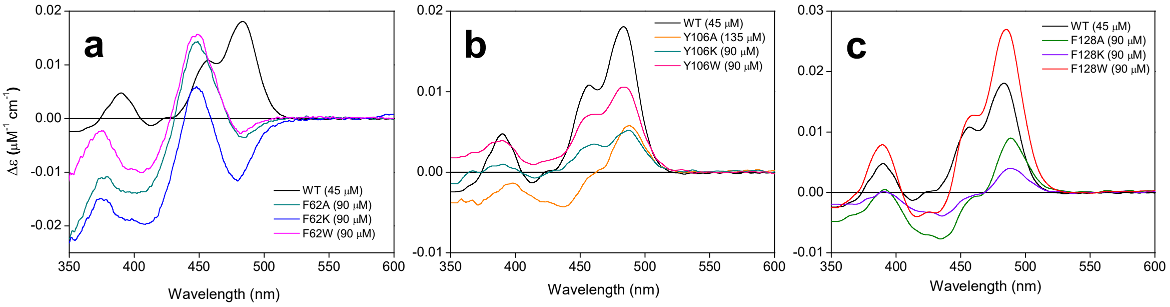


**Figure S3.** Internalization of the isoalloxazine ring of FMN in *Ca*FADS variants. Visible difference spectra elicited with (**a**) F62, (**b**) Y106 and (**c**) F128 *Ca*FADS variants (4–6 μM) upon titration with saturating FMN concentrations (indicated in parenthesis for each variant). In all panels the difference spectrum of WT is included for comparison. Spectra recorded in 20 mM PIPES, 10 mM $MgCl_2$, pH 7.0 at 25 °C.

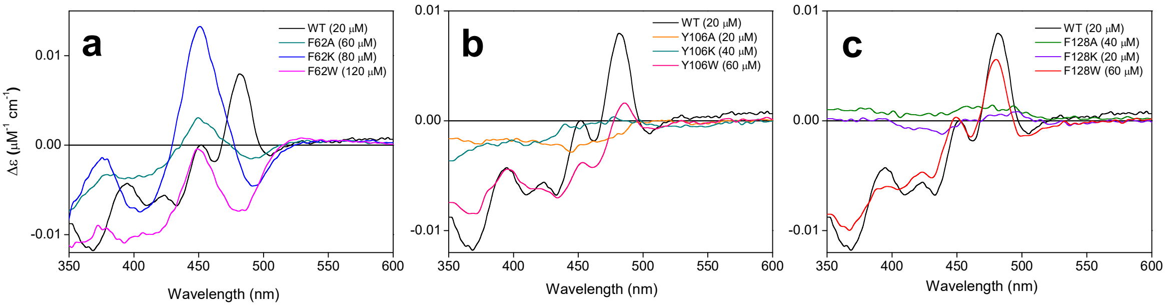


**Figure S4.** Internalization of the isoalloxazine ring of FAD in *Ca*FADS variants. Visible difference spectra elicited with (**a**) F62, (**b**) Y106 and (**c**) F128 variants *Ca*FADS (4–6 μM) upon titration with saturating FAD concentrations (indicated in parenthesis for each variant). In all panels the difference spectrum of WT is included for comparison. Spectra recorded in 20 mM PIPES, 10 mM $MgCl_2$, pH 7.0 at 25 °C.

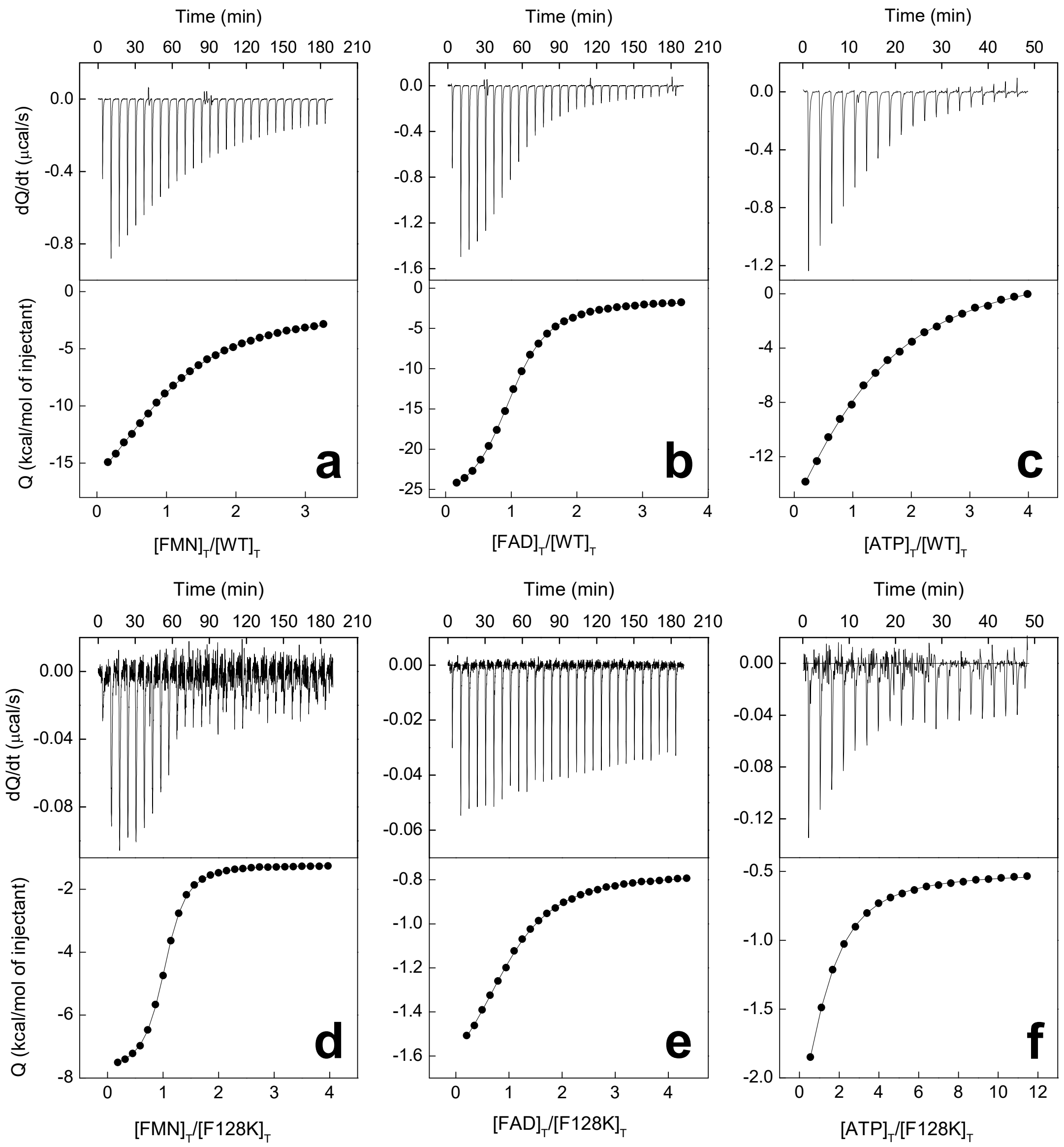


**Figure S5.** Binding affinity of ligands to WT and F128K *Ca*FADS variants. Thermogram (*upper panels*) and binding isotherms with integrated heat (*lower panels*) for the titration of WT (**a**–**c**) and F128K (**d**–**f**) with FMN (**a** and **d**), FAD (**b** and **e**) and ATP (**c** and **f**) in 20 mM PIPES, pH 7.0 at 25 ºC. Titrations performed with 10 mM $MgCl_2$ for FMN and FAD and in its absence for ATP.

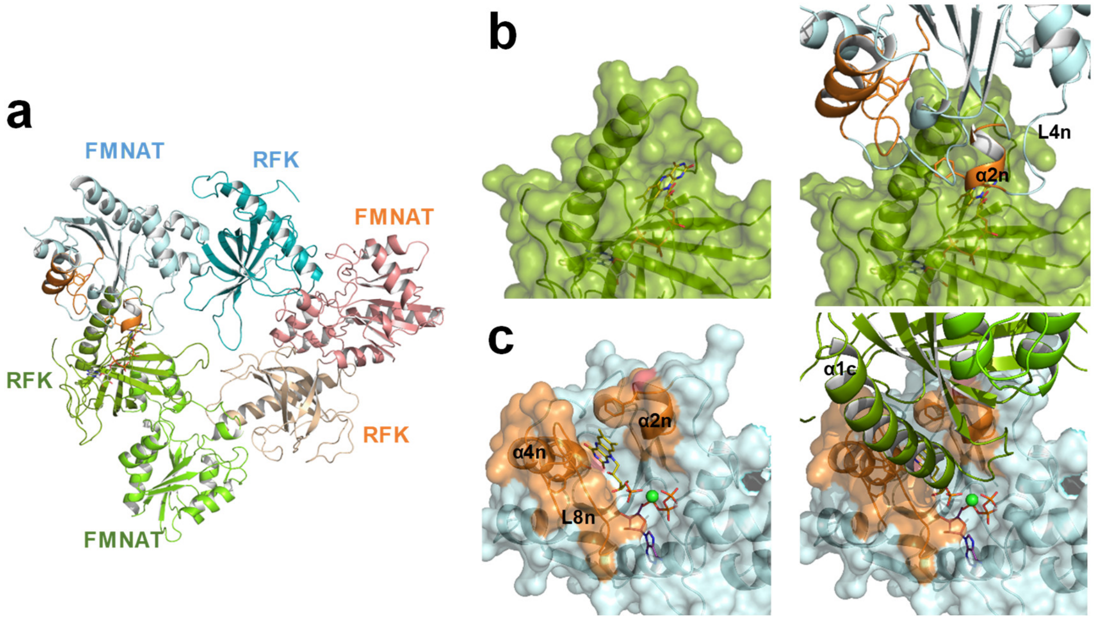


**Figure S6.** Quaternary assembly of *Ca*FADS (PDB 2x0k). (**a**) Cartoon representation of one of the trimers of the dimer-of-trimers assembly of *Ca*FADS. (**b**) Surface representation of the RFK active site at the RFK module of one monomer (*left*) and of the approaching of the FMNAT module of the neighbor protomer within the trimer (*right*). (**c**) Surface representation of the FMNAT active site cleft at the FMNAT domain of one monomer (*left*) and of the approaching of the RFK module of the neighbor protomer within the trimer (*right*). The structural elements containing residues F62, Y106 and F128 at the FMNAT module are colored in orange.

## SUPPLEMENTARY TABLES

**Table S1.** Extinction coefficients for the *Ca*FADS variants. Values determined at 279 nm in 20 mM PIPES, 10 mM $MgCl_2$, pH 7.0 at 25 ºC, *n*=3, mean ± SD.

| | **$\varepsilon^{279}$ in Gdn/HCl[a] ($mM^{-1}cm^{-1}$)** | **$\varepsilon^{279}$ in PIPES ($mM^{-1}cm^{-1}$)** | **$(\varepsilon^{279}_{PIPES} - \varepsilon^{279}_{Gdn/HCl})/\varepsilon^{279}_{PIPES}$ (%)** | **$\varepsilon^{279} - \varepsilon^{279}_{WT}$** |
|---|---|---|---|---|
| WT | 27.4 | 27.5 ± 0.2 | 0.4 | -- |
| F62A | 27.4 | 28.2 ± 0.3 | 2.8 | 0.7 |
| F62K | 27.4 | 28.6 ± 0.2 | 4.2 | 1.1 |
| F62W | 33.1 | 34.9 ± 0.1 | 5.2 | 7.4 |
| Y106A | 26.2 | 29.2 ± 0.2 | 10.3 | 1.7 |
| Y106K | 26.2 | 34.6 ± 0.1 | 24.3 | 7.1 |
| Y106W | 33.1 | 34.4 ± 0.3 | 3.8 | 6.9 |
| F128A | 27.4 | 29.9 ± 0.2 | 8.4 | 2.4 |
| F128K | 27.4 | 29.7 ± 0.2 | 7.7 | 2.2 |
| F128W | 33.1 | 35.9 ± 0.1 | 7.8 | 8.4 |

[a] Theoretical value based on the aminoacid sequence (Gill and von Hippel 1989).

**Table S2.** Thermodynamic parameters for the interaction of *Ca*FADS variants with flavin and adenine nucleotides. Values were determined in 20 mM PIPES, 10 mM $MgCl_2$, pH 7.0 for FMN and FAD and in 20 mM PIPES, pH 7.0 for ATP, at 25 ºC, $n$=3, mean ± SD.

| | Ligand | Δ*H* (kcal/mol) | Δ*G* (kcal/mol) | -TΔ*S* (kcal/mol) |
|---|---|---|---|---|
| WT | FMN | −22 ± 1 | −7.0 ± 0.1 | 15 ± 1 |
| | FAD | −26 ± 1 | −8.1 ± 0.1 | 18 ± 1 |
| | ATP | −44 ± 6 | −5.9 ± 0.1 | 39 ± 6 |
| F62A | FMN | −1.6 ± 0.1 | −6.6 ± 0.1 | −5.0 ± 0.1 |
| | FAD | −0.4 ± 0.1 | −7.0 ± 0.1 | −6.5 ± 0.1 |
| | ATP | −16 ± 2 | −5.9 ± 0.1 | 11 ± 3 |
| F62K | FMN | −1.8 ± 0.1 | −7.2 ± 0.1 | −5.4 ± 0.1 |
| | FAD | −11 ± 1 | −5.6 ± 0.1 | 4.8 ± 0.6 |
| | ATP | −29 ± 5 | −5.8 ± 0.1 | 23 ± 5 |
| F62W | FMN | −0.4 ± 0.1 | −8.3 ± 0.1 | −8.0 ± 0.1 |
| | FAD | −33 ± 1 | −7.2± 0.1 | 25 ± 1 |
| | ATP | −43 ± 9 | −5.6 ± 0.1 | 37 ± 9 |
| Y106A | FMN | −2.3 ± 0.1 | −8.1 ± 0.1 | −5.8 ± 0.1 |
| | FAD | −0.7 ± 0.1 | −7.6 ± 0.1 | −7.0 ± 0.1 |
| | ATP | −43 ± 6 | −6.2 ± 0.1 | 37 ± 6 |
| Y106K | FMN | −0.9 ± 0.1 | −9.0 ± 0.1 | −8.0 ± 0.1 |
| | FAD | −0.9 ± 0.1 | −8.0 ± 0.1 | −7.1 ± 0.1 |
| | ATP | −28 ± 11 | −5.8 ± 0.1 | 22 ± 11 |
| Y106W | FMN | −3.5 ± 0.1 | −7.9 ± 0.1 | −4.3 ± 0.1 |
| | FAD | −21 ± 1 | −7.1 ± 0.1 | 14 ± 1 |
| | ATP | −51 ± 7 | −5.9 ± 0.1 | 45 ± 7 |
| F128A | FMN | −1.0 ± 0.1 | −8.8 ± 0.1 | −7.8 ± 0.1 |
| | FAD | −1.0 ± 0.1 | −6.6 ± 0.1 | −5.6 ± 0.1 |
| | ATP | −4.6 ± 0.7 | −6.1 ± 0.1 | −1.5 ± 0.7 |
| F128K | FMN | −6.6 ± 0.1 | −9.3 ± 0.1 | −2.6 ± 0.1 |
| | FAD | −1.1 ± 0.1 | −7.8 ± 0.1 | −6.7 ± 0.1 |
| | ATP | −3.6 ± 0.2 | −6.6 ± 0.1 | 3.0 ± 0.2 |
| F128W | FMN | −1.7 ± 0.1 | −9.0 ± 0.1 | −7.3 ± 0.1 |
| | FAD | −60 ± 2 | −7.6 ± 0.1 | 52 ± 2 |
| | ATP | −69 ± 5 | −6.4 ± 0.1 | 62 ± 5 |

# REFERENCES

Gill, S. C. and P. H. von Hippel. Calculation of protein extinction coefficients from amino acid sequence data. *Anal. Biochem.* **1989**, *182(2)*, 319–326.
